# Thoracic epidural anesthesia improves outcomes in patients undergoing cardiac surgery: meta-analysis of randomized controlled trials

**DOI:** 10.1186/s40001-015-0091-y

**Published:** 2015-03-15

**Authors:** Shengsuo Zhang, Xinmin Wu, Hang Guo, Li Ma

**Affiliations:** Department of anesthesiology, General Hospital of Beijing military region PLA, Beijing, 100010 China; Department of anesthesiology, The First Hospital, Peking University, Beijing, 100034 China

**Keywords:** Epidural anesthesia, Cardiac surgery, Cardiac function, Meta-analysis

## Abstract

To assess the efficacy of thoracic epidural anesthesia (TEA) with or without general anesthesia (GA) versus GA in patients who underwent cardiac surgery, PubMed, Embase, the Cochrane online database, and Web of Science were searched with the limit of randomized controlled trials (RCTs) relevant to ‘thoracic epidural anesthesia’ and ‘cardiac surgery’. Studies were identified and data were extracted by two reviewers independently. The quality of included studies was also assessed according to the Cochrane handbook. Outcomes of mortality, cardiac and respiratory functions, and treatment-associated complications were pooled and analyzed. The comprehensive search yielded 2,230 citations, and 25 of them enrolling 3,062 participants were included according to the inclusion criteria. Compared with GA alone, patients received TEA and GA showed reduced risks of death, myocardial infarction, and stroke, though there were no significant differences (*P* > 0.05). With regard to treatment-related complications, the pooled results for respiratory complications (risk ratio (RR), 0.69; 95% CI: 0.51, 0.91, *P* < 0.05), supraventricular arrhythmias (RR, 0.61; 95% CI: 0.42, 0.87, *P <* 0.05), and pain (mean difference (MD), −1.27; 95% CI: −2.20, −0.35, *P* < 0.05) were 0.69, 0.61, and −1.27, respectively. TEA was also associated with significant reduction of stays in intensive care unit (MD, −2.36; 95% CI: −4.20, −0.52, *P <* 0.05) and hospital (MD, −1.51; 95% CI: −3.03, 0.02, *P* > 0.05) and time to tracheal extubation (MD, −2.06; 95% CI:−2.68, −1.45, *P* < 0.05). TEA could reduce the risk of complications such as supraventricular arrhythmias, stays in hospital or intensive care unit, and time to tracheal extubation in patients who experienced cardiac surgery.

**Electronic supplementary material:**

The online version of this article (doi:10.1186/s40001-015-0091-y) contains supplementary material, which is available to authorized users.

## Review

### Introduction

The evolution of techniques and knowledge of anesthesiology has resulted in decreased occurrence of surgery-related complications and subsequently improved clinical outcomes after surgery [1-3]. General anesthesia (GA) and thoracic epidural anesthesia (TEA) have been introduced in cardiac and pulmonary surgery for a long time [4]. It is suggested that TEA could provide better outcomes after operation than GA [5-7]. Indeed, lots of studies have focused on this issue and reported that TEA was associated with better outcomes and less operation-related complications [8-10].

As TEA has the potential of perfect pain control and high satisfaction within patients, it is a highly effective procedure for relieving acute pain after operation or severe trauma of the chest [11,12]. Studies also reveal that TEA has the advantages of improving myocardial oxygen balance, increasing coronary perfusion, and reducing complications such as supraventricular arrhythmias after surgery [13,14]. In addition, TEA may also reduce the duration of tracheal intubation and stay in intensive care unit and thus may save the patients with appropriate cost-effectiveness [15,16]. However, the application of TEA in clinical practice is more or less limited because of its increased risk of adverse events such as epidural hematoma or abscess, even spinal cord compression [10,17]. Besides, required systemic anticoagulation during cardiac surgery may promote the happening of epidural hematoma relevant to the use of an epidural catheter [18]. With regard to these concerns, the efficacy of TEA is controversial. It is crucial to update the advantages and disadvantages of TEA in the treatment of patients who have experienced surgery.

Patients (1,178) were identified in a previously published meta-analysis and it showed that the risk of death or myocardial infarction after cardiac surgery was similar between patients treated with TEA versus GA, but TEA treatment was associated with a less incidence of respiratory complications and dysrhythmias than GA alone [19]. However, since then, more randomized controlled trials (RCTs) comparing the effect of TEA either in combination with GA or alone versus GA alone have appeared in the database [14,20].

In this meta-analysis, we aimed to address whether TEA with or without GA in cardiac surgery could improve the clinical outcomes such as mortality and reduce cardiac, pulmonary, or neurological complications, expecting to provide reliable evidence in determining the risk-benefit ratio of TEA.

### Methods

#### Data sources and search strategy

By combining well-selected synonyms for cardiac surgery and epidural anesthesia, pertinent articles were retrieved by two reviewers comparing TEA with or without GA versus GA alone from PubMed, Embase, the Cochrane online library, Web of Science, with restrictions for English language and RCT type. The function of ‘see related articles’ in PubMed was used to complement additional citations. In addition, if the full text was not available, we contacted authors for a complete manuscript. The detailed PubMed and Embase search strategy was developed according to Vesna Svircevic *et al.* [14] and can be obtained from Additional file 1.

#### Study selection

Studies identified from systematic searches of database and literatures were initially reviewed at the title or abstract level by two authors independently, and disagreements were resolved by discussion or a third reviewer. With regard to the rest references, we defined eligibility criteria to further select related citations and these criteria were as follows: the ages of patients should not be less than 18; randomization was used to allocate patients to treatment; studies containing the comparison of TEA with or without GA versus GA alone, and no restriction in dose and administration of GA; studies comparing cardiac surgery with or without spinal anesthesia were excluded; animal experimental researches, limited data of interest in studies, and duplicate literatures were also discarded. Studies with limited data were excluded and one hundred percentage of agreement on included studies were tried to make.

#### Data extraction and quality evaluation

The primary outcomes were mortality and myocardial infarction, and the secondary outcomes were pulmonary complications (pneumonia or limited function of respiratory), cardiac complications (supraventricular dysrhythmias or other events), and neurological complications (hematoma or abscess in epidural, transient ischaemic attack and others). Information relevant to the above outcomes as well as baseline data were abstracted by two well-trained reviewers independently. Other data such as duration of mechanical ventilation, hospital stay, and indicators of heart function and injury were also collected. As the mortality data in individual studies were reported at different times of follow-up, we defined and measured the death rate at short-term and long-term after operation.

The Cochrane Handbook for systematic reviews of interventions (version 5.1) [21] was introduced to appraise the internal validity of eligible studies and assess the risk of bias of selection, allocation, performance, detection, and reporting in each selected article. The whole evaluation process was done by one reviewer and checked by another investigator. If a consensus decision was encountered and this was not disappeared by discussion, then the third author was involved in the final decision.

#### Data synthesis and analysis

The overall effect of TEA was assessed on the improvement of primary and secondary outcomes based on the data of included RCTs. The dichotomous variables in individual studies were presented as risk ratio (RR) with a 95% confidence interval (CI) or odds ratio (OR) with a 95% CI. The review manager 5.2 software was used to perform quantitative analysis. As ordinarily accepted, the fixed effects model for pooled analysis was firstly used.

Heterogeneity across eligible studies was detected by using the *I*^2^ statistic, which is a quantitative measure of lack of consistency across studies. If an *I*^2^ statistic of studies is between 0% to 50%, it is considered that low heterogeneity exists within these studies, those with an *I*^2^ statistic of 50% to 75% are considered to have moderate heterogeneity, and if *I*^2^ statistic is larger than 75%, a high degree of heterogeneity are considered in these trials [22]. Usually, it is considered that there is no important heterogeneity if the value of *I*^2^ across the studies is less than 50% [23]. When significant heterogeneity was detected, a random-effects model was used for analysis. Subgroup or sensitivity analyses were applied if there was a necessity.

### Results

#### Search results

**Figure 1:**
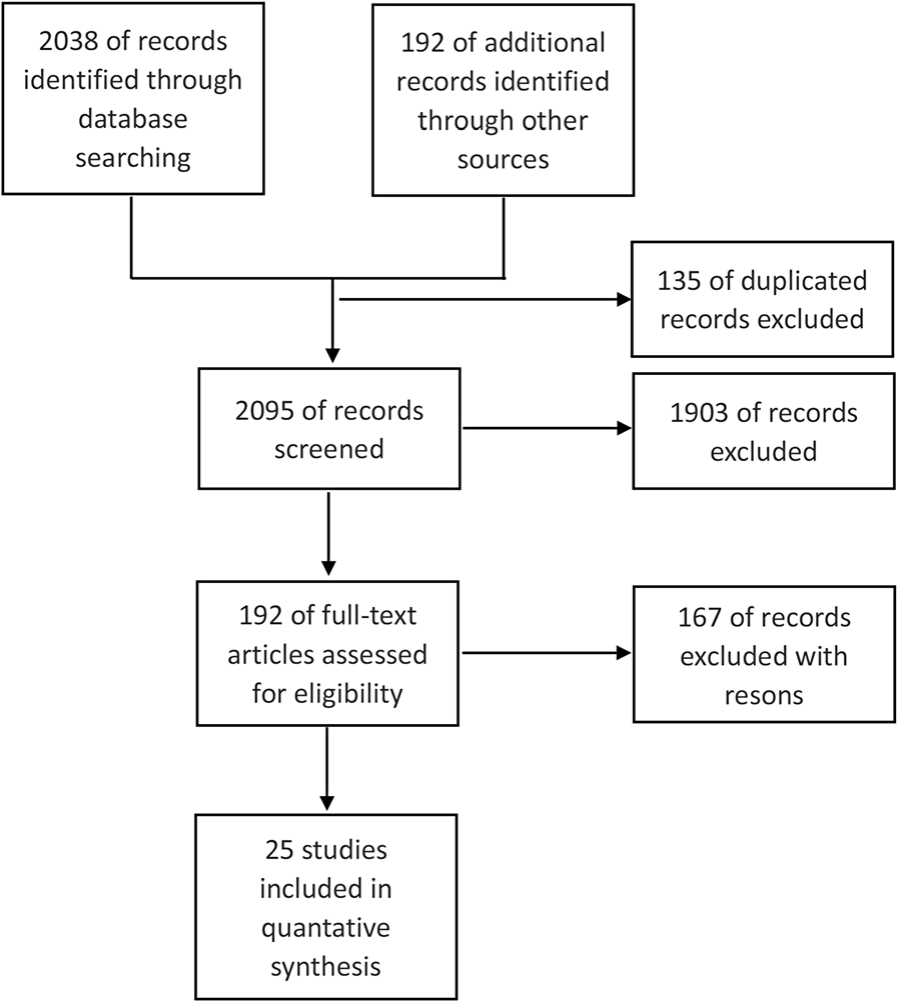
**Study flow diagram.**

A total of 2,230 articles were initially identified by the comprehensive search. One hundred and thirty five of them were discarded as they were duplicative references, and 1,903 of them were excluded after a careful review of their titles or abstracts. The assessment of full text further excluded 130 articles as they failed to meet the eligibility criteria. For the remaining 35 articles, there were no experimental design in one study, no outcome data (further detailed information could not be obtained via contacting authors) in 5 articles, and these citations were also excluded. Eventually, twenty-five RCTs [24-49] were included and their data were extracted and synthesized to evaluate the combining effect of TEA on clinical outcomes. The detailed process of study selection was illustrated in Figure 1 and the characteristics of eligible studies were presented in Table 1.

**Table 1:** **Baseline characteristics of included studies**

**First author**	**Year**	**Patients (** ***n*** **)**		**Age (mean ± std)**		**Sex(M/F)**		**Surgery**	**TEA medication**
		**TEA ± GA**	**GA**	**TEA ± GA**	**GA**	**TEA ± GA**	**GA**		
Bach	2002	13	13	60 ± 8	64 ± 7	11/2	17/10	ECABG	Bupivacaine
Bakhtiary	2007	66	66	66 ± 8	64 ± 9	54/12	58/8	OCBG	Ropivacaine and sufentanil
Barrington	2005	60	60	63 ± 9	62 ± 10	53/7	51/9	CABG	Ropivacaine/fentanyl
Berendes	2003	36	37	61 ± 11	59 ± 12	11/26	9/27	ECABG	Bupivacaine and sufentanil
Caputo	2009	36	38	63.8 ± 9.8	66.5 ± 9.3	32/4	34/4	OCBG	Propofol and fentanyl
Caputo	2011	109	117	65.9 ± 8.8	65.5 ± 8.6	102/7	102/15	OCBG	Bupivacaine
de Vries	2002	30	60	57 ± 11	60 ± 11	20/10	47/13	OPCABG	Bupivacaine and sufentanil
Fillinger	2002	30	30	-	-	-	-	CABG	Bupivacaine/ morphine
Hansdottir	2006	55	55	65 ± 10	68 ± 11	38/20	38/17	ECS	Bupivacaine
Heijmans	2007	15	45	61 ± 10	-	-	-	ECS	Bupivacaine and remifentanil
Kendall	2004	10	20	66 ± 4.6	-	8/2	14/6	ECS	Isoflurane and bupivacaine
Lagunilla	2006	25	25	66.08 ± 8.28	64.04 ± 10.15	22/3	22/3	ECS	Ropivacaine/fentanyl
Lundstrom	2005	26	24	66 ± 15	63 ± 11	-	-	ECS	Bupivacaine
Nygard	2004	79	84	-	-	-	-	CABG	Bupivacaine
Onan	2011	15	15	58.5 ± 6.0	59.4 ± 9.3	14/1	13/2	CABG	Bupivacaine
Priestley	2002	50	50	58 ± 10	60 ± 8	8/42	6/44	CABG	Ropivacaine/fentanyl
Scott	2001	206	202	59.2 ± 8.94	58.8 ± 9.18	-	-	CABG	Bupivacaine
Svircevic	2011	325	329	65 ± 10	64 ± 10	266/59	277/52	CABG	
Kiliçkan	2006	40	40	61.1 ± 8.9	58.6 ± 13.1	16/4	17/3	CABG	Bupivacaine/fentanyl
Royse	2003	37	39	64.2 ± 9.3	65.1 ± 10.8	30/7	30/9	CABG	Ropivacaine/fentanyl
Sharma	2010	30	30	58.2 ± 8.0	58.0 ± 8.3	27/3	29/1	OPCABG	Bupivacaine/fentanyl
Gurses	2013	32	32	62.8 ± 10.5	61.7 ± 8.8	8/24	10/22	CABG	Fentanyl/ levobupivacaine
Rajakaruna	2013	109	117	65.9 ± 8.8	65.5 ± 8.6	102/7	102/15	OPCABG	Bupivacaine
Jakobsen	2012	30	30	70.9 ± 4.6	71.6 ± 4.5	21/9	18/12	ECS	Bupivacaine
Onan	2013	20	20	59.2 ± 9.6	58.1 ± 6.7	19/1	17/3	CABG	Bupivacaine

*Abbreviation*: *TEA* thoracic epidural anesthesia, *GA* general anesthesia, *ECABG* elective arterial coronary bypass grafting, *OCBG* off-pump coronary bypass grafting, *CABG* arterial coronary bypass grafting, *OPCABG* off-pump CABG surgery, *ECS* elective cardiac surgery.

#### Risk of bias in included studies

**Figure 2:**
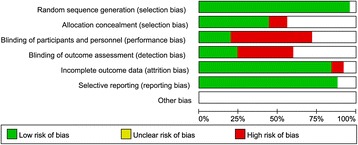
**Risk of bias of included studies based on the authors’ judgement.**

**Figure 3:**
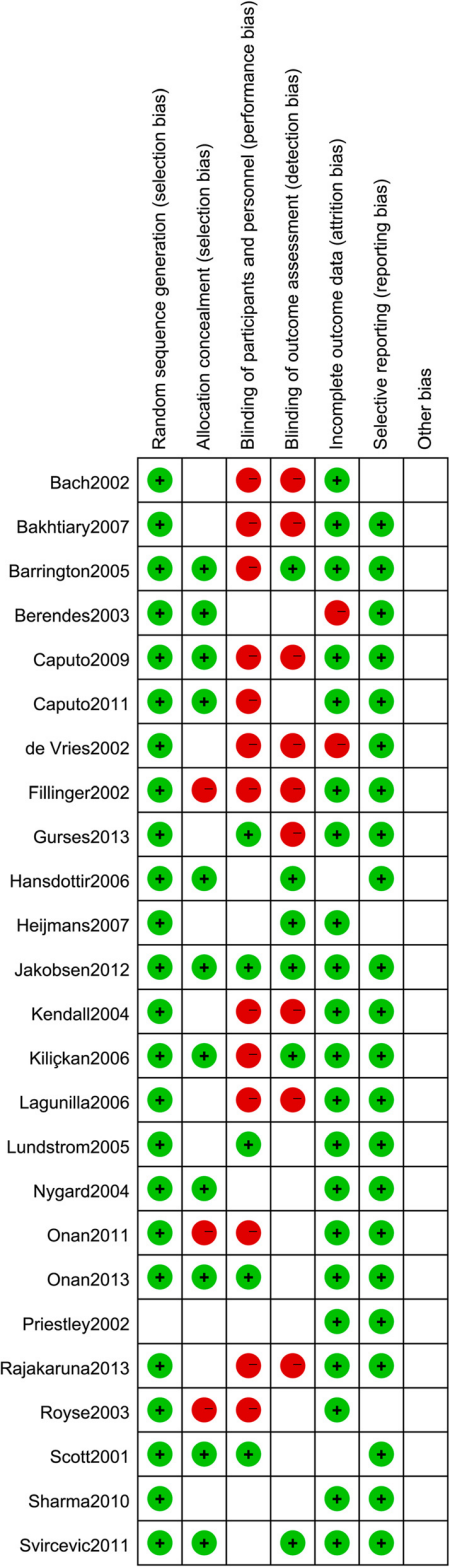
**Risk of bias summary.**

**Figure 4:**
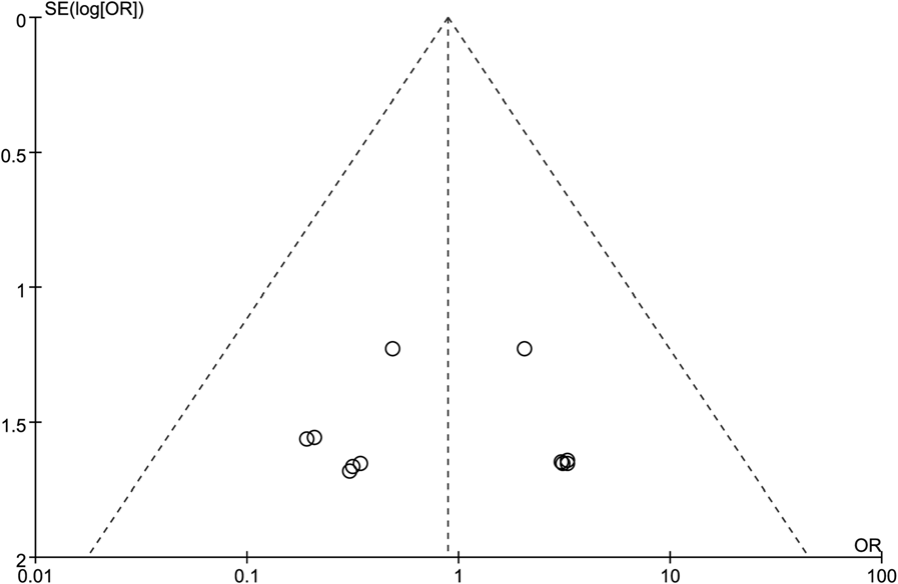
**Funnel plot of included studies relevant to mortality.**

We mainly assessed the risk of bias of selection, performance, detection, attrition, and reporting. Although we only included randomized controlled trials, the selection bias remained unclear due to the incomplete reporting of randomization and allocation method. As illustrated in Figures 2 and 3, the application of blinding was reported in a few articles and the majority of the included studies were with high risk of performance bias. With regard to the bias of attrition and reporting, the risks were relatively low. The funnel plot showed that there was no significant publication bias (Figure 4).

#### Effects of interventions

##### Mortality

**Figure 5:**
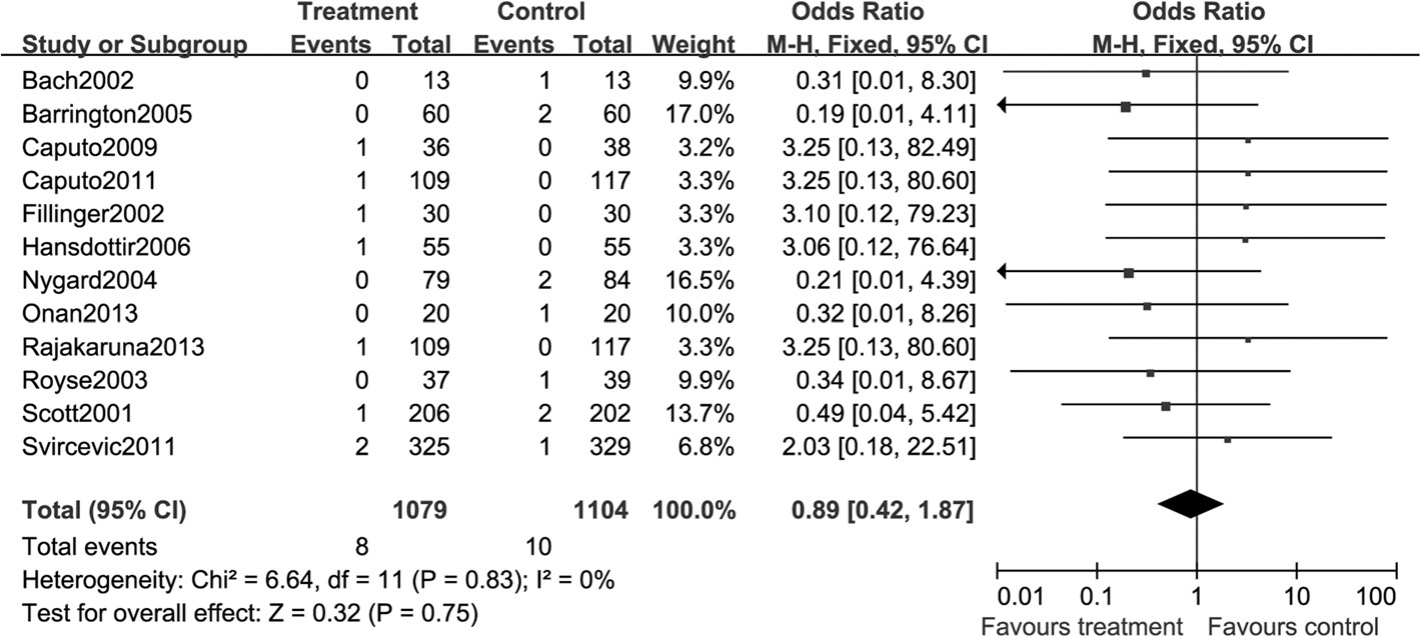
**A Forest plot for the comparison of epidural anesthesia versus control on the pooled endpoint death.**

Among the eligible studies, 12 of them provided the data of mortality. In these RCTs, the TEA intervention was not associated with a significant improvement in mortality. Death in either TEA group or GA group was infrequent. A total of 8 deaths were presented in TEA group and 10 events were reported in GA group. At last, these studies enrolling 2,181 patients were included for the combined analysis. As the measured heterogeneity was not significant, we selected fixed-effects model to perform the analysis. As presented in Figure 5, the application of TEA had an effect on reducing the risk of death with an estimate RR of 0.89, but it was not statistically significant either in short term or in long term (RR, 0.89; 95% CI:0.42, 1.87. *P* > 0.05).

##### Myocardial infarction

**Figure 6:**
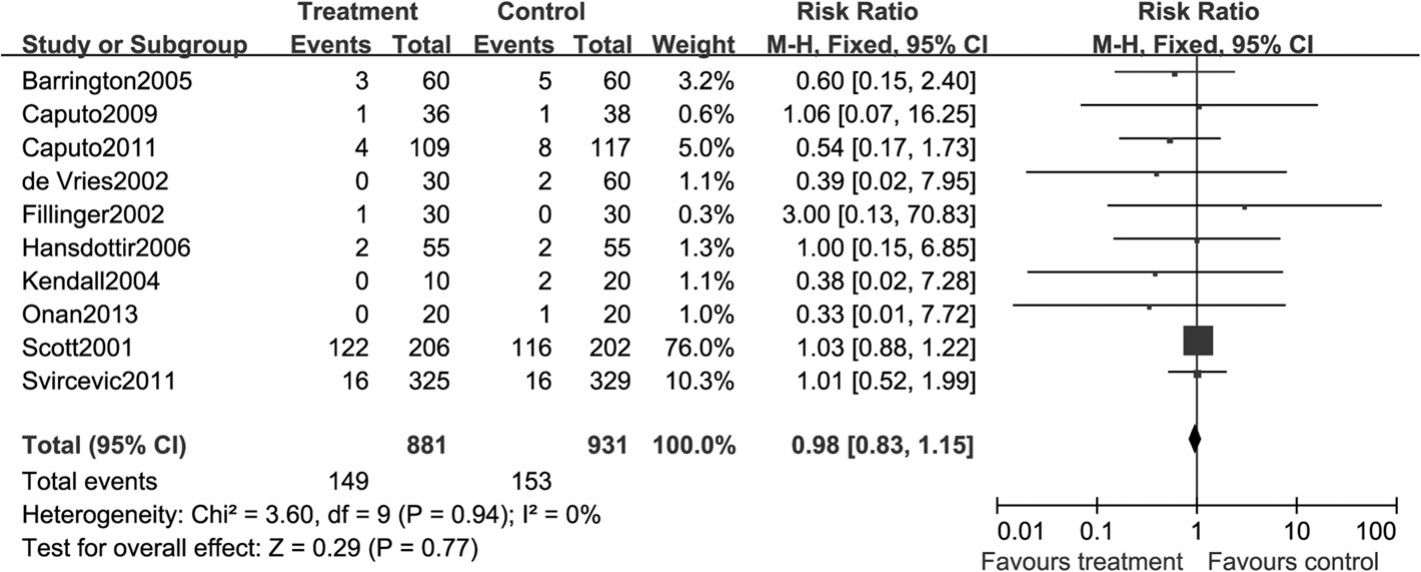
**Meta-analysis of the effect of epidural anesthesia versus control on the composite endpoint myocardial infarction.**

The pooled analysis of effect of TEA on the myocardial infarction was performed using the data from ten articles with 1,812 participants. A total of 149 events were reported in the TEA intervention, compared to the GA group with 153 events. As the value of *I*^2^ was less than 20% and the value of *Z* was larger than 0.1, we did not find a significant heterogeneity. Figure 6 shows the synthesized result and exhibits that the TEA treatment was not sufficient to significantly prevent the patients from suffering myocardial infarction, compared with GA alone (RR, 0.98; 95% CI:0.83, 1.15. *P* > 0.05). Sensitivity analysis did not show a significant effect on the results.

##### Pain relief

**Figure 7:**
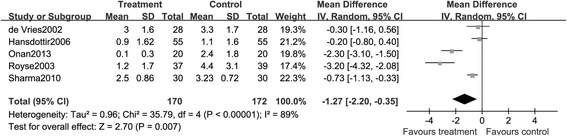
**Summarized comparison of epidural anesthesia versus control on the pain relief.**

There were eight articles which reported the effect of TEA on pain control, and most of them used the visual analog scale (VAS) score to determine the degree of pain. We used fixed-effects model to perform this analysis and found that patients received TEA suffered less pain than those of GA treatment (mean difference, −1.27; 95% CI: −2.20, −0.35, *P* < 0.05. Figure 7).

##### Stays in hospital or intensive care unit

**Figure 8:**
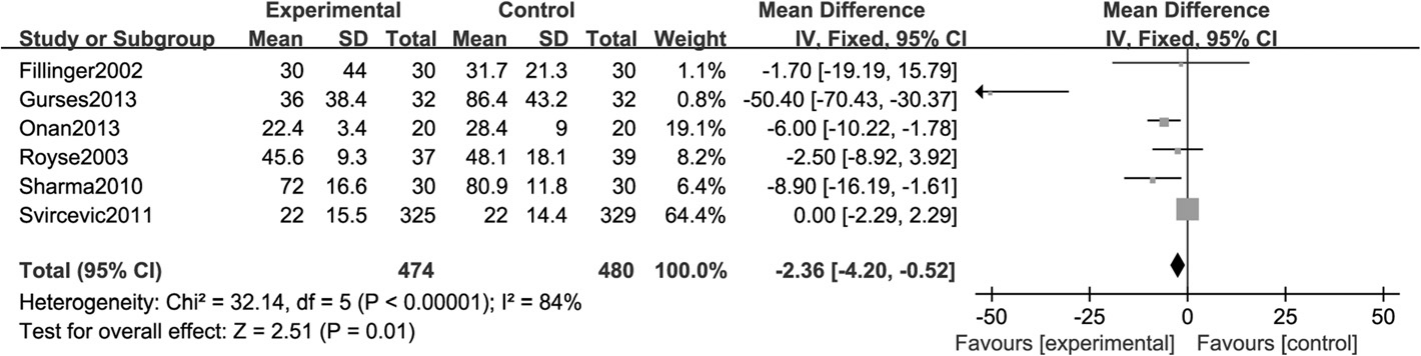
**A Forest plot for the comparison of epidural anesthesia versus control on the outcome of stays in intensive care unit.**

**Figure 9:**
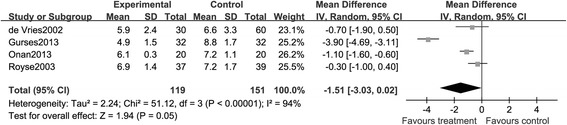
**A Forest plot for the comparison of epidural anesthesia versus control on the stays in hospital.**

A total of eight studies provided information on stays in hospital or intensive care unit; however, the duration of stay and terms of reporting varied between studies, increasing the risk of inconsistency. The pooled analysis exhibited that the use of TEA significantly reduced the time spent in intensive care unit (MD, −2.36; 95% CI: −4.20, −0.52, *P <* 0.05. Figure 8) and hospital (MD, −1.51; 95% CI: −3.03, 0.02, *P* > 0.05. Figure 9), indicating a relatively lower cost.

##### Time to tracheal extubation

**Figure 10:**
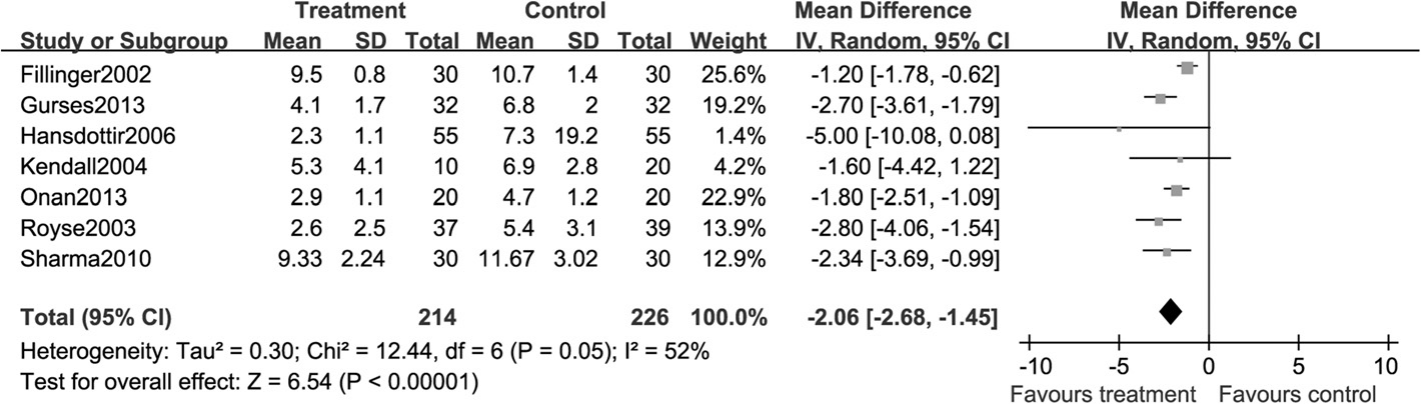
**A Forest plot for the comparison of epidural anesthesia versus control on the composite endpoint time to tracheal extubation.**

The time to tracheal extubation was reported in seven studies, and the reporting term and unit of time were different among these trials. By checking the value of *I*^2^, we found there was no significant heterogeneity in studies. As illustrated by Figure 10, compared to GA arm, TEA arm showed a significant reduction of time to tracheal extubation (MD, −2.06; 95% CI: −2.68, −1.45. *P* < 0.05).

##### Supraventricular tachyarrhythmias

**Figure 11:**
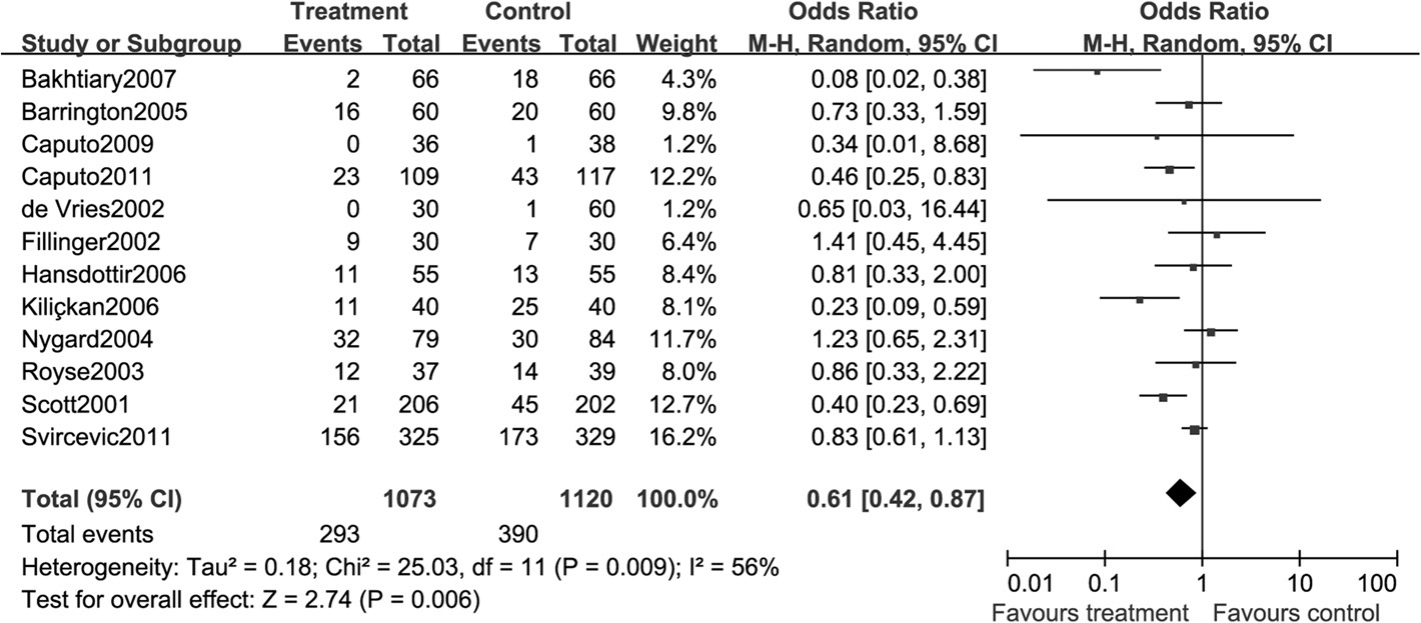
**A meta-analysis of the comparison of epidural anesthesia versus control on the supraventricular tachyarrhythmias.**

Overall, 2,193 patients from 12 RCTs were included in this pooled analysis, and there were 293 events in the TEA group and 390 events in the GA group. The detection of heterogeneity showed a low inconsistency across the studies and thus the fixed-effects model was applied. The result of meta-analysis indicated that there was no significant effect on the prevention of supraventricular tachyarrhythmias by adding TEA to GA (RR, 0.61; 95% CI: 0.42, 0.87, *P <* 0.05. Figure 11).

##### Respiratory complications

**Figure 12:**
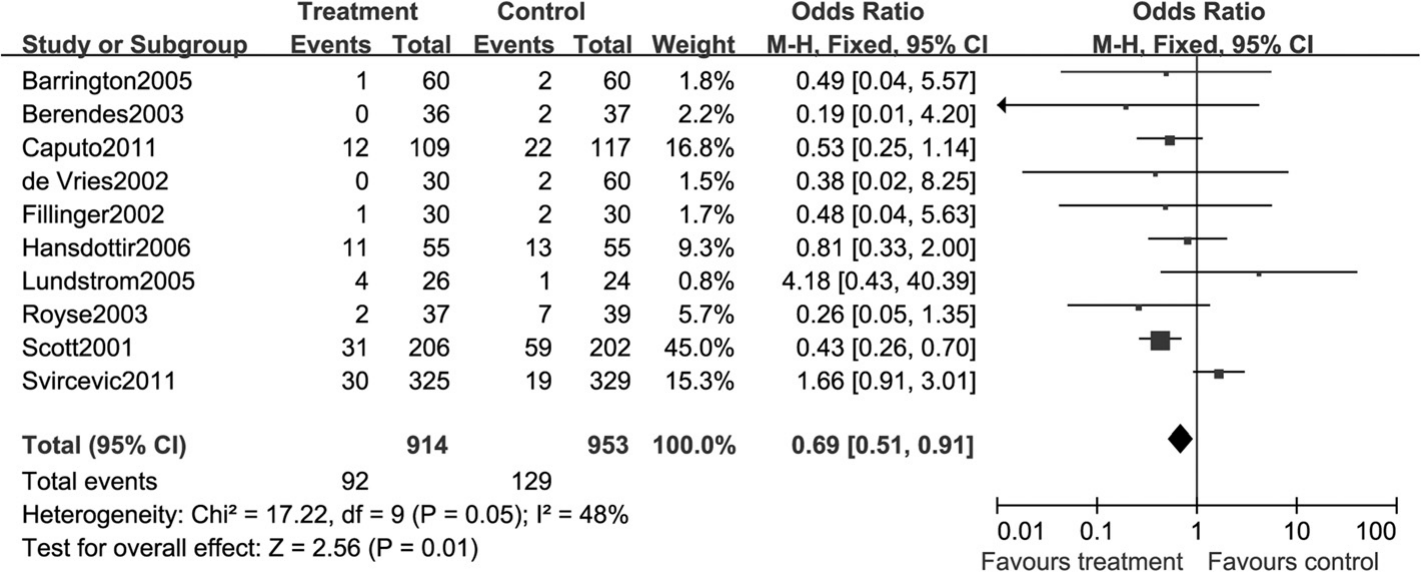
**A Forest plot for the comparison of epidural anesthesia versus control on the composite endpoint respiratory complications.**

With regard to the respiratory complications, 10 studies with 1,867 patients reported information on the incidence of events. The majority of these complications appeared within 14 days after surgery. A total of 92 events were reported in the TEA arm and 129 events were shown in the GA arm. The heterogeneity among these trials was not significant (*I*^2^ = 48%, *P =* 0.05). By using fixed-effects model, the results illustrated by Figure 12 showed that TEA could significantly reduce the prevalence of respiratory complications for patients with cardiac surgery, compared with patients receiving GA during operation (RR, 0.69; 95% CI: 0.51, 0.91, *P* < 0.05).

##### Neurologic events

**Figure 13:**
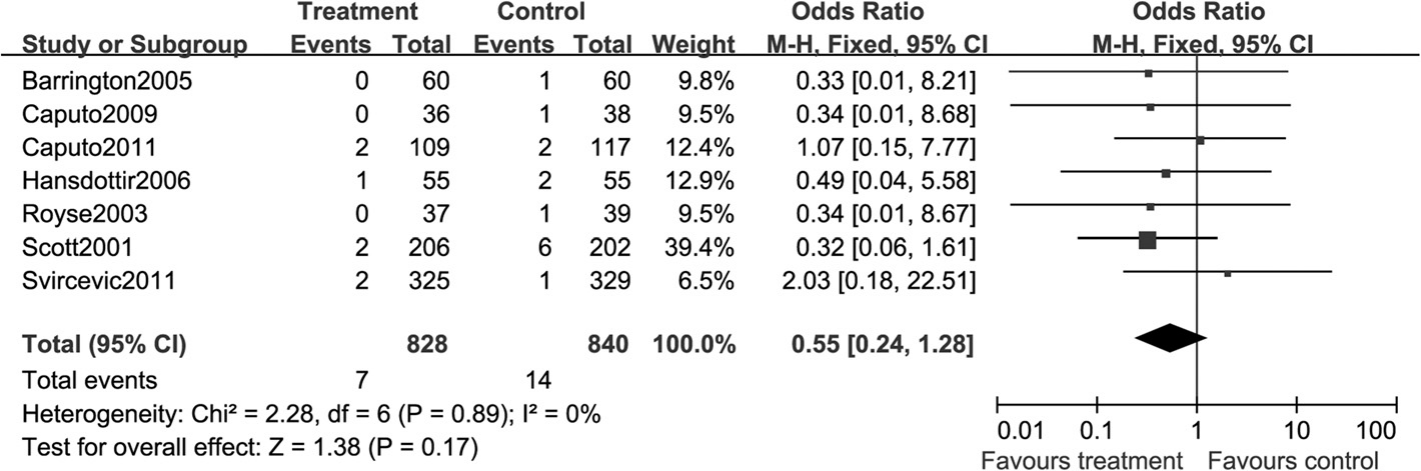
**A Forest plot for the comparison of epidural anesthesia versus control on the composite endpoint stroke.**

As TEA was reported with a higher risk of incidence of epidural hematoma or abscess, we planned to perform this analysis to determine this effect. However, these events were not reported in all of the included studies. Stroke was another rare complication and only seven RCTs reported the data of this event, making it difficult to fully reveal the effect of TEA on the incidence of stroke. The TEA group had 7 events and the GA group had 14 events. After evaluating the heterogeneity of these studies (*I*^2^ = 0%, *P* > 0.05), we performed this analysis and found that there was a lower risk of stroke in patients receiving TEA and GA, compared with those with GA alone (RR, 0.55; 95% CI: 0.24, 1.28, *P* > 0.05. Figure 13).

### Discussion

This meta-analysis using combined data from several RCTs determined that TEA with or without GA did not show a benefit in reducing the rate of death or the risk of myocardial infarction during perioperation, though TEA with or without GA showed a significant effect on reduction of risk of supraventricular tachyarrhythmias, respiratory complications, and time to extubation. These results suggested that TEA on the top of GA could provide additional benefits on clinical outcomes for patients receiving cardiac surgery. The effect of TEA on lowering the risk of mortality or myocardial infarction may be underpowered due to the extremely low events in both groups.

The methodological assessment of quality of included trials suggests that the reliability of our study is moderate and this is mainly due to the incomplete details of biases of selection, blinding and attrition. Randomized controlled trials are the optimal evidence for analyzing effect of intervention, [50] and this kind of studies are included in our meta-analysis. Though randomization has been applied in these articles, allocation concealment, which is another kind of selection bias, is rarely reported, resulting in a moderate risk of selection bias. As for biases of performance and detection, the risk is relatively high due to the unclear information about blinding in each study, and this is the main cause of the hampered quality of our study. There are also other biases caused by incomplete outcome data or selective reporting; however, these biases are associated with low risk and have little effect on the overall heterogeneity. Considering the imperfect quality of included trials, we carefully give our recommendation on the finding of this meta-analysis.

TEA is considered as the gold standard analgesic intervention for major surgery [51]. TEA with or without GA has the potential to provide sufficient pain control and has been used in cardiac surgery for years. Mortality as a severe result of cardiac surgery can be related to the complicated procedures or perioperative injury or pain. The activation of endocrine, neural, and metabolic pathways and inflammatory reactions all contribute to this unfavorable event probably by damaging myocardium, causing pain and other reasons [51]. Though cardiac surgery may result in mortality, the incidence of mortality is very low. In our meta-analysis, by combining the events in both TEA arm and GA arm, we found that the rate was 0.8% (18 deaths/2,181 patients in total). The pooled result suggested that the relief of pain was significantly improved in the TEA arm, compared with GA group. But this reduction of pain did not result in a lower risk of mortality. This may be explained by the extremely low and varied reporting of mortality in included studies.

Myocardial infarction is one of the most serious complications related to surgery, making myocardium one of the most important tissues needs protection from ischemia during surgeries [52]. Although there were different definitions of myocardial infarction used in included studies, most of them correctly defined this event by employing biomarkers of myocardium and ECG examination. Myocyte necrosis/apoptosis is the main cause of elevation of biomarkers after cardiac surgery; one study [42] reported that TEA plus GA preserved cardiac function by reducing apoptosis and improving hemodynamic function. However, the combined data in our study showed that TEA and GA had a similar effect on lowering the occurrence of myocardial infarction. One of the explanations may be the direct myocardial injury from ventricular venting, sewing needles or direct cardioversion or manipulation of the heart, [52] which could not be simply protected by TEA.

This meta-analysis also suggested that application of TEA could save patients from suffering cardiac or pulmonary or neurologic complications and reducing duration of intubation and stays in intensive care unit or hospital. These may contribute to a better cost-effectiveness of TEA, despite its insufficient efficacy on reducing mortality and myocardial infarction. Studies have reported that TEA may improve myocardial oxygen balance and reduce perioperative stress response [53,54]. We did find a benefit of TEA in controlling heart beats; however, the stress response could not be assessed by meta-analysis due to the limited information provided by few studies.

There are a few similar meta-analysis published in recent years [14,19,55]. Liu *et al.* [19] found that TEA was associated with significant reduction of arrhythmias, pulmonary complications, time to tracheal intubation, and postoperative pain. Their meta-analysis failed to determine the beneficial effect of TEA on reducing risk of clinical outcome (myocardial infarction for example). Another meta-analysis published by Guay [55] suggested that in patients operated on under GA, the addition of TEA reduced the incidence of arrhythmia with an OR of 0.59, and the time to tracheal extubation was reduced by 3.9 h, stay of intensive care unit also reduced. In our meta-analysis, we suggested that among patients who experienced cardiac surgery treatment-related complications and consumption of time in hospital or intensive care unit were reduced. Cardiac functions could be improved by using TEA, although TEA could not reduce the risk of death. Our result is consistent with previous meta-analysis. Recently, Mehta Y pointed out that TEA might decrease pulmonary, cardiovascular, or renal complications, provide excellent analgesia, and allow early extubation in high-risk cardiac surgical patients [56]. A larger scale RCT should be done to further confirm this point.

As drawbacks of meta-analysis are difficult to avoid, there are also few limitations in this study, making our findings less reliable. First, the application of TEA in cardiac surgery is in a debate, and this study failed to determine a significant reduction of mortality or myocardial infarction in patients treating with TEA plus GA. There is an urgent need for large number, multi-center, randomized, blinded clinical trials which could provide sufficient data on mortality or myocardial infarction or stroke as well as epidural hematoma relevant to the intervention of TEA. Second, although all of the included studies were RCTs, the quality of them varied. There was an obvious bias of selection because most of them did not report the information of blinding. And the time point of measuring and methods of detection were not in accordance with each other. Third, some of the studies have been published for several years, and there are remarkable developments in procedures or techniques after these years, and this may contribute to a higher risk of heterogeneity when these studies were included. Finally, there may be some publication bias in this meta-analysis, which may exaggerate the difference between TEA with or without GA and GA alone in the cardiac surgery.

## Conclusions

This meta-analysis suggests that the anesthetic regimen of TEA is not sufficient to reduce the risk of mortality or myocardial infarction after cardiac surgery; however, TEA appears to benefit patients by lowering incidence of respiratory complications, cardiac events, or neurologic complications and reducing duration of postoperative ventilation. Viewing the related risk of epidural hematoma or abscess, the application of TEA should be carefully considered in well-selected patients who are going to undergo cardiac surgery, unless multi-center RCTs with large number of participants are available and their data could be powered to an outcome favoring the application of TEA.

## Additional file

Additional file 1:
**Searching strategy.** Databases.

## Abbreviations

CI: confidence interval
GA: general anesthesia
OR: odds ratio
RCTs: randomized controlled trials
RR: risk ratio
TEA: thoracic epidural anesthesia
VAS: visual analog scale
