# Supplementary material for: Thoracic epidural anesthesia improves outcomes in patients undergoing cardiac surgery: meta-analysis of randomized controlled trials
Source: Eur J Med Res. 2015 Mar 15;20(1):25. doi: 10.1186/s40001-015-0091-y (PMC4375848; doi:10.1186/s40001-015-0091-y)
Supplement: Additional file 1: — Searching strategy. Databases. [file 40001_2015_91_MOESM1_ESM.docx]

Additional file 1. Searching strategy

*Database*

PubMed and MEDLINE (1966 –2014)

*Searchfilter*

((“Cardiac Surgical Procedures”[MeSH] or cardiac surgery[tiab] or heart surgery[tiab] or cardiac surgical procedures[tiab] or cardiopulmonary bypass[tiab] or cardiothoracic*[tiab] or CABG[tiab]) not Pulmonary Surgical Procedures[MeSH]) and (“Analgesia, Epidural”[MeSH] or “Anesthesia, Epidural”[MeSH] or “Anesthesia, Spinal”[MeSH] or epidural*[tiab] or peridural*[tiab] or extradural*[tiab] or spinal*[tiab] or subarachnoid*[tiab] or intrathecal*[tiab] or neuraxial*[tiab]) and ((randomized controlled trial [pt] or controlled clinical trial [pt] or randomized controlled trials [mh] or double-blind method [mh] or singleblind method [mh] or clinical trial [pt] or clinical trials [mh] or (“clinical trial” [tw])) or ((singl* [tw] or doubl* [tw] or trebl* [tw] or tripl* [tw]) and (mask* [tw] or blind* [tw])) or (placebos [mh] or placebo* [tw] or random* [tw] or research design [mh:noexp] or comparative study [mh] or evaluation studies [mh] or follow-up studies [mh] or prospective studies [mh] or control* [tw] or prospective* [tw] or volunteer* [tw] not (animals [mh] not human [mh])))

*Database*

EMBASE (1989 –2014)

*Searchfilter*

(((heart-surgery in su) or (cardiopulmonary-bypass in su)) or ((coronary artery bypass surgery or coronary artery surgery or coronary bypass graft surgery or coronary artery bypass graft or coronary bypass graft or coronary artery bypass graft* or coronary bypass graft* or CABG or ((off pump or offpump or offpump) and (coronary surgery)) or open heart surgery or heart surgery or heart valve surgery or cardiopulmonary bypass) and ((xrec = ab) or (xrec = ti)))) and (((epidural or peridural or extradural or spinal or subarachnoid or intraspinal or intrathecal or neuraxial) and ((xrec = ab) or (xrec = ti))) or ((spinal-anesthesia or intraspinal-drug-administration or epidural-anesthesia) in su)) and (((controlled study or controlled trial or clinical study or major clinical study or clinical trial or randomized controlled trial or random* or trial*) and ((xrec =ab) or (xrec = ti))) or ((clinical study or controlled study) in su))
